# Response to HIV-1 gp160-carrying recombinant virus HSV-1 and HIV-1 VLP combined vaccine in BALB/c mice

**DOI:** 10.3389/fmicb.2023.1136664

**Published:** 2023-03-17

**Authors:** Beibei Zhang, Hongyan Mao, Hongjuan Zhu, Jingxia Guo, Paul Zhou, Zhenghai Ma

**Affiliations:** ^1^Xinjiang Key Laboratory of Biological Resources and Genetic Engineering, College of Life Science and Technology, Xinjiang University, Ürümqi, Xinjiang, China; ^2^Unit of Antiviral Immunity and Genetic Therapy, Key Laboratory of Molecular Virology and Immunology, Institut Pasteur of Shanghai, Chinese Academy of Sciences, Shanghai, China

**Keywords:** herpes simplex virus type 1 (HSV-1), HSV-1-based vector, HIV vaccine, virus-VLP boost, immune response

## Abstract

Human immunodeficiency virus (HIV) induced AIDS causes a large number of infections and deaths worldwide every year, still no vaccines are available to prevent infection. Recombinant herpes simplex virus type 1 (HSV-1) vector-based vaccines coding the target proteins of other pathogens have been widely used for disease control. Here, a recombinant virus with HIV-1 *gp160* gene integration into the internal reverse (IR) region-deleted HSV-1 vector (HSV-BAC), was obtained by bacterial artificial chromosome (BAC) technology, and its immunogenicity investigated in BALB/c mice. The result showed similar replication ability of the HSV-BAC-based recombinant virus and wild type. Furthermore, humoral and cellular immune response showed superiority of intraperitoneal (IP) administration, compared to intranasally (IN), subcutaneous (SC) and intramuscularly (IM), that evidenced by production of significant antibody and T cell responses. More importantly, in a prime-boost combination study murine model, the recombinant viruses prime followed by HIV-1 VLP boost induced stronger and broader immune responses than single virus or protein vaccination in a similar vaccination regimen. Antibody production was sufficient with huge potential for viral clearance, along with efficient T-cell activation, which were evaluated by the enzyme-linked immunosorbent assay (ELISA) and flow cytometry (FC). Overall, these findings expose the value of combining different vaccine vectors and modalities to improve immunogenicity and breadth against different HIV-1 antigens.

## Introduction

Human immunodeficiency virus type 1 (HIV-1) induced immune deficiency syndrome (AIDS) is widespread across the world and continues to rise.^1^ Since its discovery in 1983, the number of infections and deaths has already exceeded a billion, and this number is still increasing despite extensive work for drugs and vaccines development (Larijani et al., 2019; Miall et al., 2022). Upon infection, HIV-1 employs multiple immune evasion strategies that allow for its replication (Desrosiers, 1999). In addition, high genetic variability and integration into the host cell genome further enhances immune escape (Woodman and Williamson, 2009; Altfeld and Gale Jr., 2015). Antiretroviral therapy (ART) is the most prevalent and effective method to control HIV infection, but it cannot fully clear the virus, requiring life-long therapy to a chronic stage of low viremia. Its subsequent toxic and side effects of lifelong medication has a significant impact in patients life quality (Barre-Sinoussi et al., 2013; Ayele et al., 2018). Therefore, vaccine-based prophylaxis has significant advantages in cost, period and curative effect over conventional therapy after infection. However, an effective vaccine to control and reduce HIV burden remains elusive (Calarota and Weiner, 2003; Spearman, 2003; Emini and Koff, 2004) because of high mutation and viral diversity and its consequent immune escape (Desrosiers, 2004). To date, traditional inactivated virus vaccines, subunit protein vaccines, viral vector vaccines and nucleic acid vaccines have been trialed at different clinical stages. Some have failed while other are still under clinical trials, but their safety or effectiveness remains unsatisfactory (Rerks-Ngarm et al., 2009; Cafaro et al., 2015; Graziani and Angel, 2016). Multiple viral-vector based vaccine have been tested in clinical trials, such as; canary poxvirus (Rerks-Ngarm et al., 2009), adenovirus (Colby et al., 2020), alphaviruses (Egan et al., 2004), lentivirus (Uhl et al., 2002), rhabdoviruses (Ramsburg et al., 2004), and herpes simplex virus (HSV) (Kaur et al., 2007); with promising results, inducing a strong immune responses *in vivo*, with high antibody titers against different HIV-1 epitopes (Rose et al., 2001; Haglund et al., 2002; Verrier et al., 2002).

HSV is a double-stranded DNA virus and can be divided into alpha, beta and gamma subtypes. HSV-1 and -2 are two of the three human alpha-HSV that create global infections, and cause gingivitis, reproductive system infections, neonatal infection, and many other diseases (James et al., 2020). The whole genome length of HSV is 152 kb and consists of a unique covalently linked long fragment (U_L_) and a short fragment (U_S_). The terminal reverse repeats (TR_L_ and TR_S_) are located at the ends of these two genes respectively, and the junction of the two segments is the internal reverse (IR) repeats of the gene, including IR_L_ and IR_S_ (Marconi et al., 2009). Of note, about half of the viral encoded proteins are involved in replication, while the rest are nonessential genes, allowing for the deletion of the IR and replacement/insertion of target genes without impacting infectivity of the recombinant virus (pathogenicity impaired) (Wang et al., 2001; Watanabe et al., 2007; Bloom et al., 2018). Moreover, HSV has broad cellular tropism, including dendritic cells (Kruse et al., 2000; Abendroth et al., 2001; Mikloska et al., 2001), which elicits strong and durable immune responses in various routes of administration (Kuklin et al., 1998; Brehm et al., 1999; Hocknell et al., 2002). Finally, HSV viral DNA persists inside the host’s cell nucleus in an episomal form, avoiding any concerns about safety and random integration into the host’s DNA. Finally, the viral genome can carry the thymidine kinase (TK) gene that can be combined with specific antiretroviral drugs to kill virus-carrying cells (Whitley et al., 1993; Spivack et al., 1995). So far, HSV vector-derived vaccines focused on HIV structural or core proteins tat (Nicoli et al., 2016), gp120 (Kanda et al., 2016), gag (Santos et al., 2007) and env (Duke et al., 2007). The Env precursor protein gp160 is cleaved into mature gp120 and gp41 by host enzymes to form virus particles, and is required for the assembly of viral surface structure of HIV particles, however, this target has not been properly assessed in a HSV vector system (Polpitiya Arachchige et al., 2018) neither its efficacy to elicit protective immune responses upon vaccination.

Human infection with HSV-1 is more common than HSV-2. HSV-1 infection usually causes limited disease symptoms in immunocompromised individuals, such as localized herpes, and infection can be effectively cured with ganciclovir or acyclovir (Woodman and Williamson, 2009; Graziani and Angel, 2016; James et al., 2020). The potential of HSV-1 as a gene therapy and vaccine vector has been long known. The modified vectors carry foreign genes that can persist stably for a long time *in vivo* and are expressed in large quantities upon replication, thereby inducing an effective immune response (Liu et al., 2009). The heterologous prime-boost approach using a recombinant viral vector carrying HIV genes followed by recombinant proteins showed a robust immune response, particularly cellular immune response (Excler and Kim, 2019). Subsequent boosting can be difficult due to vector-targeted neutralizing responses, but this can be overcome with a VLP protein boosts (Xiao et al., 2021). The main question is how this compares to a HIV VLP prime-boost strategy. Here, we evaluate the immune response to recombinant HSV-1 vector-based virus encoding HIV-1 *gp160* in a mouse model. Briefly, the cellular and humoral immune responses were investigated among prime-boost strategies using the virus prime followed by HIV-1 virus-like particles (VLP) boost. The finding of these potential candidate HSV-1 vaccine vectors and the way of combined immunization with HIV-1 VLP will shed light for moving on to primate challenge studies.

## Materials and methods

### Cells, plasmids, virus and proteins

Vero cells were stored in our lab and grown in Dulbecco’s Modified Eagle Medium (DMEM) containing 10% fetal bovine serum (FBS; Gibco, Carlsbad, CA, USA), 100 U/ml penicillin and 100 μg/ml streptomycin (Thermo Fisher Scientific; Waltham, MA, USA) at 37°C with 5% CO2. *Drosophila* S2 cells stably expressing HIV-1 VLP protein were constructed previously (Yang et al., 2012) and kindly provided by Dr. Paul Zhou (Institute Pasteur of Shanghai, China) and maintained in complete Express Five™ SFM medium (Thermo Fisher Scientific) at 28°C without CO2. The HSV-BAC plasmid containing full length HSV-1 17 strain (HSV-1 17; GenBank number: NC_001806.2) but lacking 15 kb of IR sequence (loss of one copy of the ICP34.5, LATs and three IE genes, ICP0, ICP4 and ICP22) in *E.coli* cells were kept in our lab.

The recombinant plasmid pcDNA3.1-gp160 carries the foreign genes from HIV-1. The plasmid pKO5-BN with cloning site containing the recombination arm upstream and downstream of IR, was selected as a shuttle plasmid vector for carrying the inserted *gp160* and bring it to the same cell environment of HSV-BAC, to produce recombinant virus through BAC-induced homologous recombination. The MH1001 (mutant HSV 1001) is an engineered HSV-1 virus derived from HSV-BAC plasmid after transfection and screening from Vero cells. All plasmids were obtained from Dr. Paul Zhou.

Recombinant HSV-1 gD protein was purchased from Abcam (Cambridge, UK). While gp120 was obtained based on previous work (Hollister et al., 2014). A Readytoprocess WAVE 25 biological reaction system (GE Healthcare; Boston, USA) was used to grow *Drosophila* S2 cells for HIV-1 VLP protein production as previously described in our lab with some minor adjustments (Mao et al., 2016). Specifically, 1.2 × 10^7^ S2 cells were seeded in a 2-liter bag with 300 mL SFM medium for 3 days culture. Subsequently, 500 mL fresh medium were added to continue culture (after 10^7^ cells/ml was reached). Six days after the initial culture, another 200 ml fresh medium was added, supplemented with 5 μM CdCl2 to induce HIV-1 VLPs. Four days after induction, the culture supernatant was harvested by centrifugation at 12,000× *g* for 30 min at 4°C and Cd^2+^ chelated with 1 mM EDTA. Subsequently, supernatants were filtered through a 0.45 mm pore size filter (Millipore, Burlington, MA, USA) and transferred to the QuixStand Benchtop system (GE Healthcare) with molecular retention of 300,000 NMWC for volumetric concentration. The 200 mL concentrated eluate was diluted with 800 mL PBS (pH 7.2 ~ 7.4) buffer and concentrated again as described above. This process was repeated three more times, and the final 200 mL PBS eluate containing HIV-1 VLPs was further concentrated with an ultrafiltration concentrator tube of 50 kDa molecular cutoff by centrifugation at 4000 rpm at 4°C until to a final volume of approximately 10 mL. Finally, HIV-1 VLP purity was assessed by 10% SDS-PAGE, followed by total protein content estimation with Pierce™ BCA Protein Assay Kit (Thermo Fisher Scientific) according to the manufacturer’s instructions.

### Construction of recombinant HSV-1 viruses expressing HIV-1 gp160 gene

The *gp160* gene was obtained through PCR with PrimeSTAR® GXL DNA Polymerase as amplification enzymes (Takara, Dalian, China), and using pcDNA3.1-gp160 as a template, according to the manufacturer’s instructions PCR products were then cloned into the downstream of pCMV vector, followed by construction of pKO5-BN-gp160, quality control checked by digestion and sequencing. The recombinant plasmids were electroporated into *E.coli* cells for homologous recombination as previously described (Horsburgh et al., 1999; Kuroda et al., 2006). Subsequently, recombinant DNAs were transfected into Vero cells with Lipofectamine® LTX & Plus Reagent (Thermo Fisher Scientific) according to the manufacturer’s instructions; and the virus was purified by three rounds of limiting dilution plaque assay. Finally, the recombinant HSV-1 viral DNAs were verified by Southern blot, and the expression of envelope proteins was detected by Western blot and Flow cytometry. The primer pairs used in the PCR are listed in Table 1.

**Table 1:** Primer pairs used to construct the recombinant virus in this study.

Name	5′–3′ (sense)
EF1 P1	AGTCCCCGAGAAGTTGGG
EF2 P2	TCACGACACCTGAAATGGAAGAA
BGH polyA P1	CTGTGCCTTCTAGTTGCC
BGH polyA P2	GTCGCCGCCGGTGATCCATAGAGCCCACCGCAT
gp160 infusion P1	GAATTCGATTTCGATATCGTTGACATTGATTATTGACTAGTTATTAATAG
gp160 infusion P2	GTCGCCGCCGGTGATATCCCATAGAGCCCACCGCAT

### Ethics statement and animal experiments

Adult female BALB/c mice (6 ~ 8 weeks of age) were purchased from Animal Laboratory Center, Xinjiang Medical University (Urumqi, Xinjiang, China) and fed with sterile water and food *ad libitum* under a 12 h light/dark cycle condition. All animal experiments in this study were approved by the Committee on Ethical Use of Animals of Xinjiang University and the care of animals was conducted strictly according to the guidelines of Xinjiang University Institutional Committee. All efforts were made to minimize animal suffering. All mice were acclimated for 1 week before immunization. All blood samples from the immunized mice were collected through the inferior ocular sinus with 0.5 mm medical glass capillary (Heqi Glassware Co., Ltd., Shanghai, China), processed for serum and stored at −80°C until use.

To optimize the administration route for HSV-1-gp160 vaccine, 30 mice were randomly divided into five inoculation groups (*n* = 6): intranasally (IN), subcutaneous (SC), intramuscular (IM), and intraperitoneally (IP). Mock saline injection was used as negative control. All mice were immunized with 100 μL 1 × 10^7^ pfu purified recombinant virus expect saline negative control. All animals were boosted with the same dose of viruses or the same volume of saline 28 days after priming immunization. After identifying the best immunization route, another 24 mice were randomly divided into four inoculation groups (*n* = 6) to set the best immunization combination; virus only (HSV-gp160 + HSV-gp160), virus+VLP (HSVgp160 + HIV-VLP), VLP only (HIV-VLP + HIV-VLP) and saline only (saline+saline), named as IP-2, IP-HV, VLP-2 and NC group, respectively. Mice were then immunized with 100 μL 1 × 10^7^ of the virus, VLP mixture containing 50 μL protein (0.1 mg/mL) and 50 μL c-diGMP adjuvant (Sigma-Aldrich, St. Louis MO, USA), or control mixture with same volume of saline and adjuvant as VLP mixture, respectively. Animals were boosted 28 after priming immunization with the same dose and volume of virus, protein or saline.

### DNA extraction and Southern blot analysis

Cells were collected by centrifugation at 3000 rpm at 4°C for 5 min and washed twice with ice-cold PBS buffer. DNA extraction was performed with DNAzol regent (Takara) according to the manufacturer’s instructions. DNA concentration and purity were determined using Epoch-BioTek Microplate Reader based on the OD_260nm_/OD_280nm_ value (1.8 ~ 2.0). MH1001-infected cells and mock-infected cells were used as the negative and blank control, respectively.

Purified DNA was digested with *Eco*R I (Takara) at 37°C overnight, then separated with the 1% agarose gel and blotted onto nitrocellulose (NC, Millipore) membrane. Subsequently, the membrane was probed with DIG-labeled DNA probes for confirmation of gp160 gene into the viral genome using DIG DNA Labeling and detection Kit (Roche, Basel, Switzerland) according to the manufacturer’s instructions. Finally, the band signal was captured using the ChemiDoc MP Image System (Bio-Rad).

### Evaluation of HIV-1 gp160 protein expression manner

To verify the total HIV-1 gp160 protein expression, Vero cells were seeded in 6-well plates at a concentration of 1 × 10^6^/mL and allowed to adhere overnight. Cells were then infected with recombinant virus or with MH1001 (MOI = 0.5) as a negative control. Cells were then harvested at indicated time points post-infection and lysed with SDS-PAGE sample buffer (1×) (Beyotime Biotechnology, Beijing, China) containing 10% beta-mercaptoethanol (β-ME). The cell lysate was separated on 12% SDS-PAGE and proteins electrotransferred to polyvinylidene difluoride membranes (PVDF, Millipore), followed by blocking with TBST buffer (Beyotime Biotechnology) containing 5% nonfat powdered milk for 2 h at 37°C. The membranes were probed with the 1:2000 diluted anti-gp120 mouse monoclonal antibody (Sino Biological Inc., Beijing, China), or 1:5000 diluted anti-β-tubulin rabbit monoclonal antibody (Beyotime Biotechnology) as the primary antibody for 2 h at room temperature (RT), followed by incubation with 1: 5000 diluted HRP-conjugated goat anti-mouse antibodies or HRP-conjugated goat anti-rabbit antibodies (TransGen Biotech, Beijing, China) as the secondary antibody for 1 h at RT. Finally, colorimetric reaction of the band was performed using ECL Western blot Substrate (Solarbio, Beijing, China), and visualized with ChemiDoc MP Image System (Bio-Rad).

For further analysis of the gp160 protein in the cell surface, flow cytometry was performed as previously described (Li et al., 2016). Briefly, virus-infected cells (2 × 10^4^) were harvested by centrifugation at 500× *g* for 5 min at 4°C and washed once with ice-cold PBS buffer. Cells were then resuspended and fixed with 100 μL 4% formaldehyde at RT for 15 min, the liquid was removed by centrifugation-washing recycle procedures twice, and cell pellets were resuspended and immunolabeled with 100 μL PBS diluted mouse anti-gp120 antibody (1:250 dilution; Jackson ImmunoResearch, West Grove, PA, USA) at RT for 1 h. Cells were washed again, and then stained with 100 μl PBS diluted FICT-conjugated goat anti-mouse antibody (H + L) (1: 800 dilution; Jackson ImmunoResearch) at RT for 30 min. Cells were washed again and resuspend in 300 μL of PBS. Finally, the samples were passed through a 300-mesh copper mesh and analyzed on a FACSCalibur (BD Biosciences, New Jersey, USA). Mock-treated and MH1001-infected cells were selected as blank and negative control, respectively, for flow gating. While double-click the stacked histogram in the Layout editor, and select the option in Y axis as % of Max in the Graph Definition window, resulting in the generation of a Y axis with relative number axis rather than the previous absolute cell count, so as to eliminate the differences caused by different cell numbers. All data analyses were conducted with the FlowJo platform (Tree Star, Inc., Ashland, OR, USA).

### Intracellular staining and flow cytometry analysis

On day 56 after the first injection, immunized mice were euthanized with 1.5 g/kg barbiturates (Sigma-Aldrich) through IP injection, and splenocytes were prepared as previously described (Yang et al., 2012). For intracellular cytokine staining (ICS) assay, 2 × 10^6^/mL splenocytes were seeded into a 24-well plate (Thermo Fisher Scientific) with complete 1,640 medium containing 10% FBS, 100 U/ml penicillin and 100 μg/ml streptomycin, and stimulated with mixtures of peptides (2 μg/ml of each peptide), containing peptides derived from HIV-1 envelope protein, along with 5 μg of anti-mouse CD28 (Abcam) and anti-mouse CD49d (Abcam). Cells were incubated for 6 h and Golgi Plug (BD Biosciences) added during the final 4 h of incubation. Cells were stained with anti-mouse CD16/32 (Fc block) antibody (ProteinTech, Wuhan, China), followed by cell surface staining with PerCP-conjugated anti-CD4 and APC-conjugated anti-CD8 antibodies (Abcam). Subsequently, cells were fixed, permeabilized with cytofix cytoperm (BD Biosciences) and stained with FITC-conjugated anti-IFN-γ (Santa Cruz Biotechnology, California, USA). Finally, 1 × 10^6^ cells per sample were analyzed on an LSR II flow cytometer (BD Biosciences) and the data subsequently analyzed using the FlowJo platform.

### Enzyme-linked immunosorbent assay (ELISA)

To evaluate the total IgG, IgG1, IgG2a and IgG3 antibody responses against HIV-1 gp120 in immunized mice, 96-well ELISA plates were coated overnight at 4°C with 100 ng/well recombinant soluble HIV-1 gp120 protein, washed with PBST buffer (Beyotime Biotechnology) and blocked at 37°C for 2 h with PBST containing 5% nonfat powdered milk the next day. After washing, sera were diluted at defined dilution (1,10^3^ or 1:10^5^) and added to wells in triplicate; and incubated for 2 h at 37°C. Plates were washed and HRP Goat anti-mouse total IgG (TransGen Biotech) or IgG1, IgG2a, and IgG3a (Sigma-Aldrich) added, followed by incubation for 1 h at RT. After 3 washes with PBST, reactivity was detected with TMB substrate (Sigma-Aldrich) and color development measured by absorbance at 450 nm by an Epoch-BioTek Microplate Reader (Winusky, Vermont, USA). The endpoint antibody levels were determined as being the reciprocal of the highest dilution of serum that had thrice the absorbance value of the pre-immune sera at the same dilution.

Sera were tested for anti-HSV-1 IgG antibodies by ELISA as described above with HSV-1 gD protein, coated plates (200 ng per well).

### Statistical analysis

All data are presented as the mean ± SD. The scatter diagram of antibody levels within each group was performed using GraphPad Prism version 7.0 (GraphPad Software, San Diego, CA, USA). Statistical significance was determined using a one-way analysis of variance (ANOVA) followed by Tukey’s multiple comparison test. The two-tailed *p* value <0.05 was considered statistically significant.

## Results

### Generation of HSV-1 recombinant virus expressing HIV-1 gp160 protein

**Figure 1:**
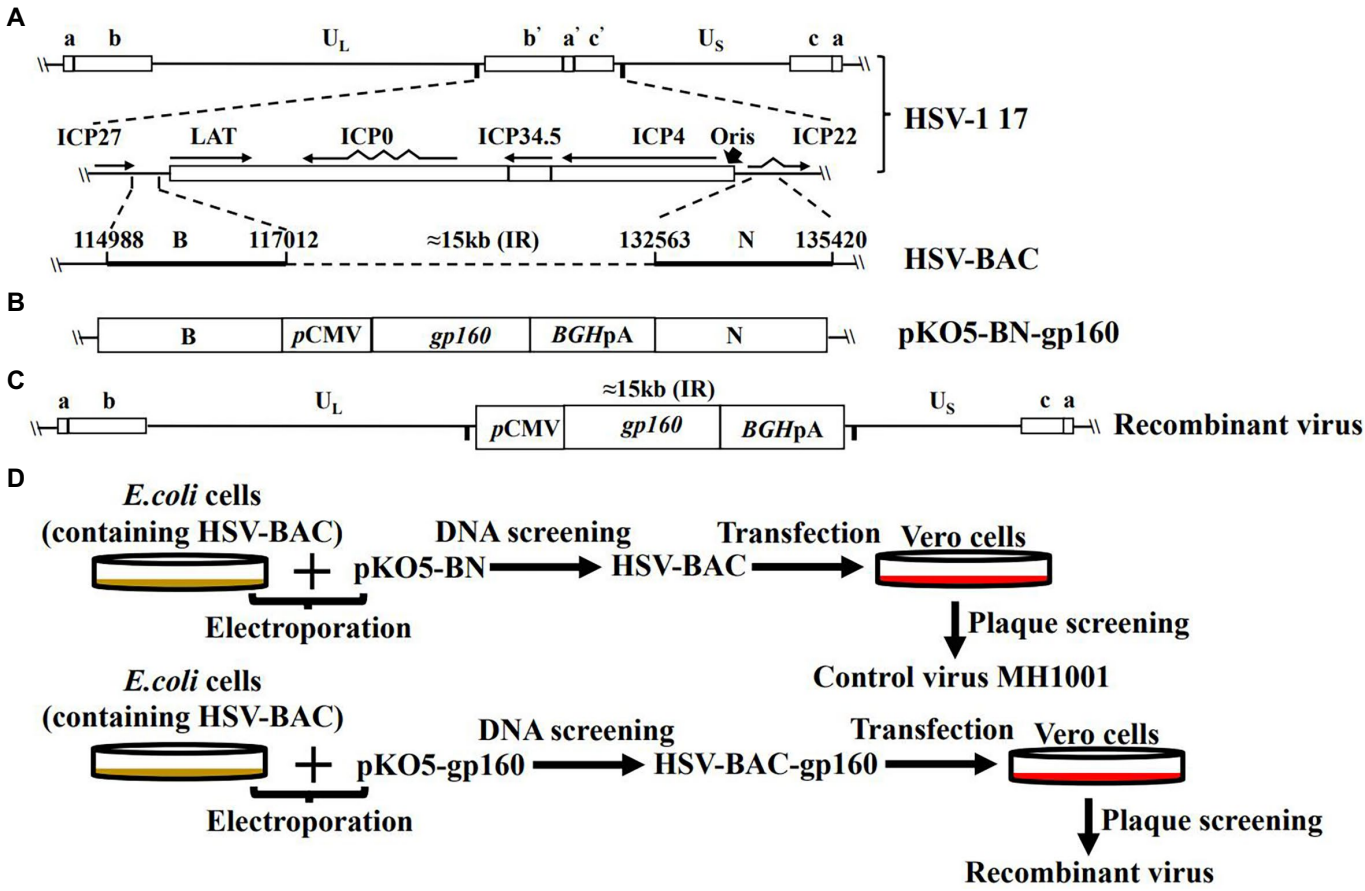
Genomic structures of HSV-1 recombinant vectors and recombinant virus constructs. **(A)** Schematic diagram of the wild-type HSV-1 genome. The HSV-1 genome consists of unique long (UL) and short (US) regions flanked by inverted repeat (IR) sequences. a, a′ = terminal repeats; b, b′ = L component inverted repeats; c, c′ = S component inverted repeats. In the HSV-1 recombinant vector, a 15-kilobase (kb) fragment, including one copy of latency-associated transcripts (LATs), ICP0I, CP34.5, ICP4, was deleted to generate HSV-BAC. **(B)** Schematic diagram of composition of pKO5-BN-gp160. The recombinant shuttle plasmid vector was obtained after insertion of gp160 sequences into the cloning site through recombination of the upstream and downstream (N) arm within IR of pKO5. Then the recombinant HSV-1 virus **(C)** with *gp160* gene was acquired by BAC triggered homologous recombination between pKO5-BN-gp160 and HSV-BAC, as shown on the flowchart **(D)**. CMV, promoter/enhancer sequences of the CMV IE gene; PA, polyadenylation signal.

**Figure 2:**
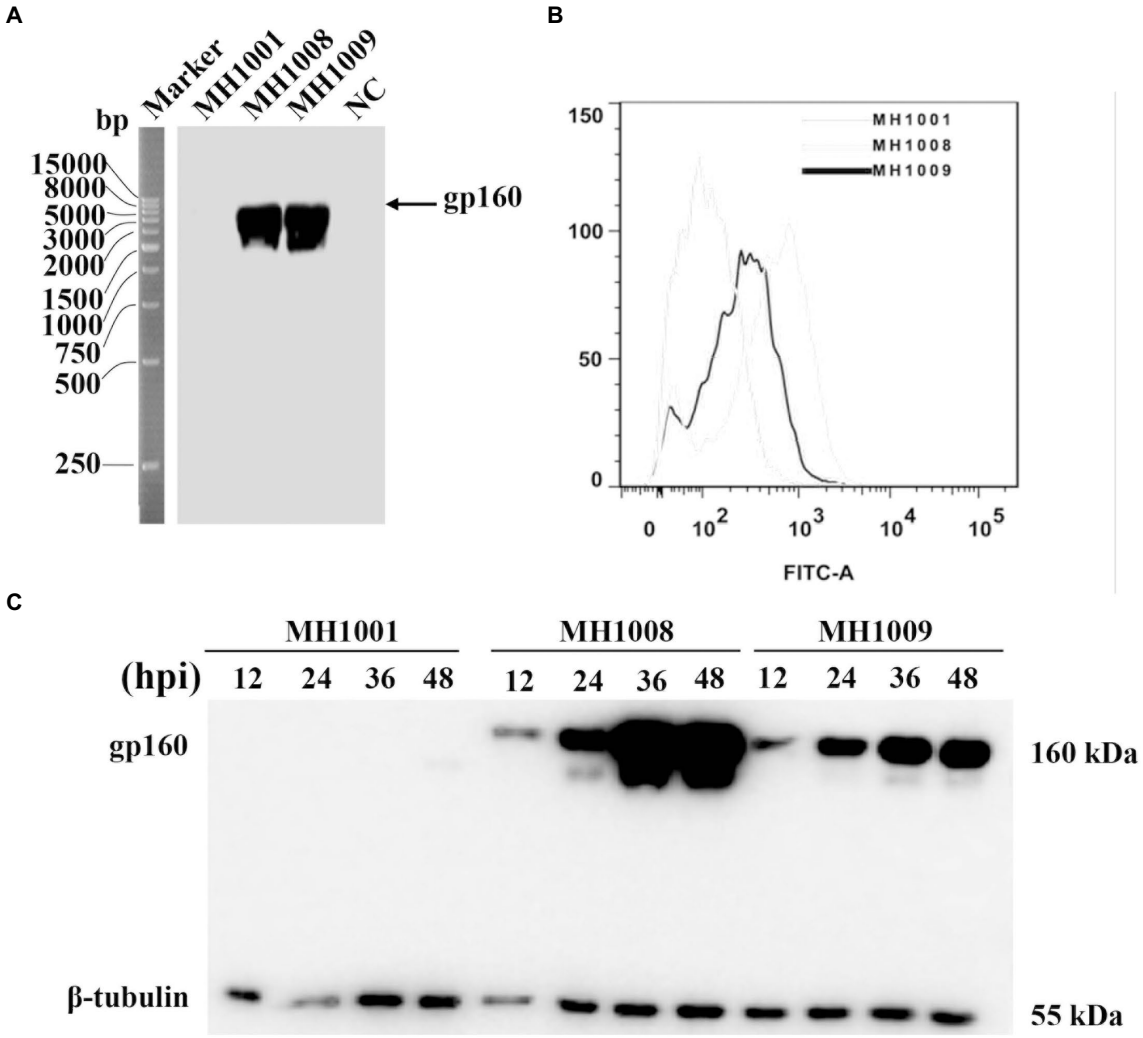
Identification and characterization of recombinant virus. **(A)** Identification of recombinant virus by Southern blot. DNA was isolated from Vero cells infected with MH1001 or recombinant virus clones (MH1008 and MH1009), followed by single enzymatic digestion. DNA hybridization was performed by Southern blot for detection of *gp160* gene in the HSV-1 genome. Mock-infected cells served as control. **(B)** Flow cytometry analysis of gp160 protein surface expression in recombinant virus-infected cells 48 h inoculation. **(C)** Time course of HIV-1 antigen expression in virus-infected cells. Vero cells were infected with viruses (MH1001, MH1008 or MH1009), and harvested at indicated timepoints for analysis of total gp160 protein expression levels by Western blot.

As illustrated in Figure 1A, a 15 kb IR sequence within the full length of HSV-1, 17 DNA sequences including one copy of ICP34.5, LATs, ICP0, ICP4 and ICP22 were deleted on the vector backbone or control virus (HSV-BAC or MH1001). The HIV-1 *gp160* gene was inserted here by homologous recombination between HSV-BAC and shuttle plasmid carrying *gp160* (pKO5-BN-gp160) (Figure 1B), to produce the recombinant virus (Figure 1C) according to the corresponding diagram (Figure 1D). Infected cell DNAs were analyzed by Southern blot to confirm the HIV-1 gene insertion into the HSV genome. The 8.9 kb fragment hybridized to a probe prepared from the HSV-1 gene and showed detectable hybridization to both HSV-gp160 virus clones MH1008 and MH1009 (Figure 2A). Evaluation of gp160 protein expression in the HSV-gp160 infected cells showed target bands with expected size, which increased up to 48 h after infection, indicating the continuous release of HIV-1 protein. The total protein level of MH1008 clone was much higher than MH1009 clone (Figure 2C), but both kept stable expression though screening process. Moreover, the cell surface expression level of gp160 protein between these two virus clones showed differences consistent with total protein (Figure 2B), thus MH1008 was selected as the candidate recombinant virus for all subsequent experiments.

### T cell responses elicited by different administration routes

**Figure 3:**
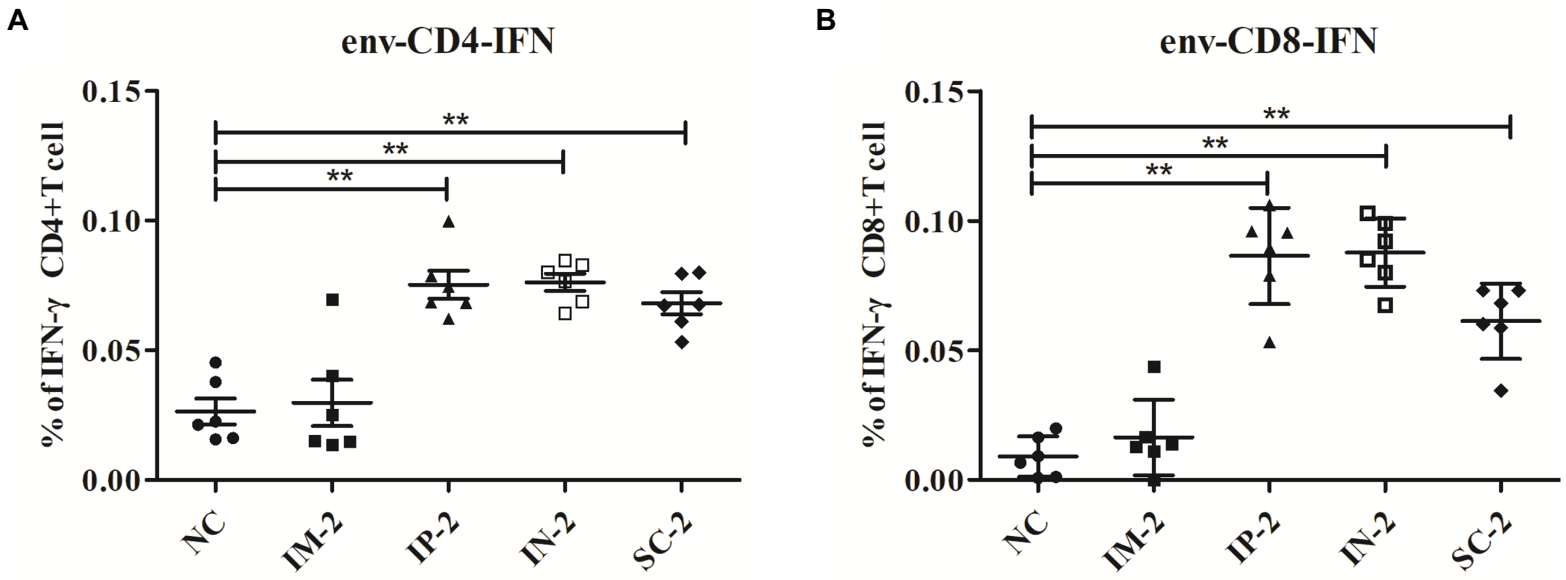
HIV-1 envelope-specific CD4 and CD8 T cell responses elicited with different administration routes. 56 days after the first injection, mice from different vaccination groups (IM, IP, IN, SC and NC group) were humanly euthanized and spleens collected. T cell responses against HIV-1 envelop-specific peptides were analyzed by flow cytometry. The percentages of activated CD4^+^ **(A)** and CD8^+^ **(B)** T cells that produce IFN-γ were detected by intracellular cytokine staining. **p < 0 0.001.

To distinguish whether different administration routes could elicit HIV-1 envelope-specific T cell responses *in vitro*, splenocytes from IM, IP, IN, SC and control mice were assessed against the envelope peptide mixtures for intracellular IFN-γ cytokine staining. CD4 and CD8 T cells of IP, IN, and SC groups all exhibited significantly higher peptide-specific responses against the envelope than the control mice (Figure 3, *p* < 0.01). Moreover, the three groups including IP, IN, and SC groups exhibited significantly higher peptide-specific CD4 and CD8 T cell responses against envelop than the IM group (*p* < 0.05). On average, the data showed that when compared to control and IM group mice, the other three groups exhibited statistically significantly better peptide-specific responses, without significant difference between them (*p* > 0.05).

### B cell responses indued by different administration routes

**Figure 4:**
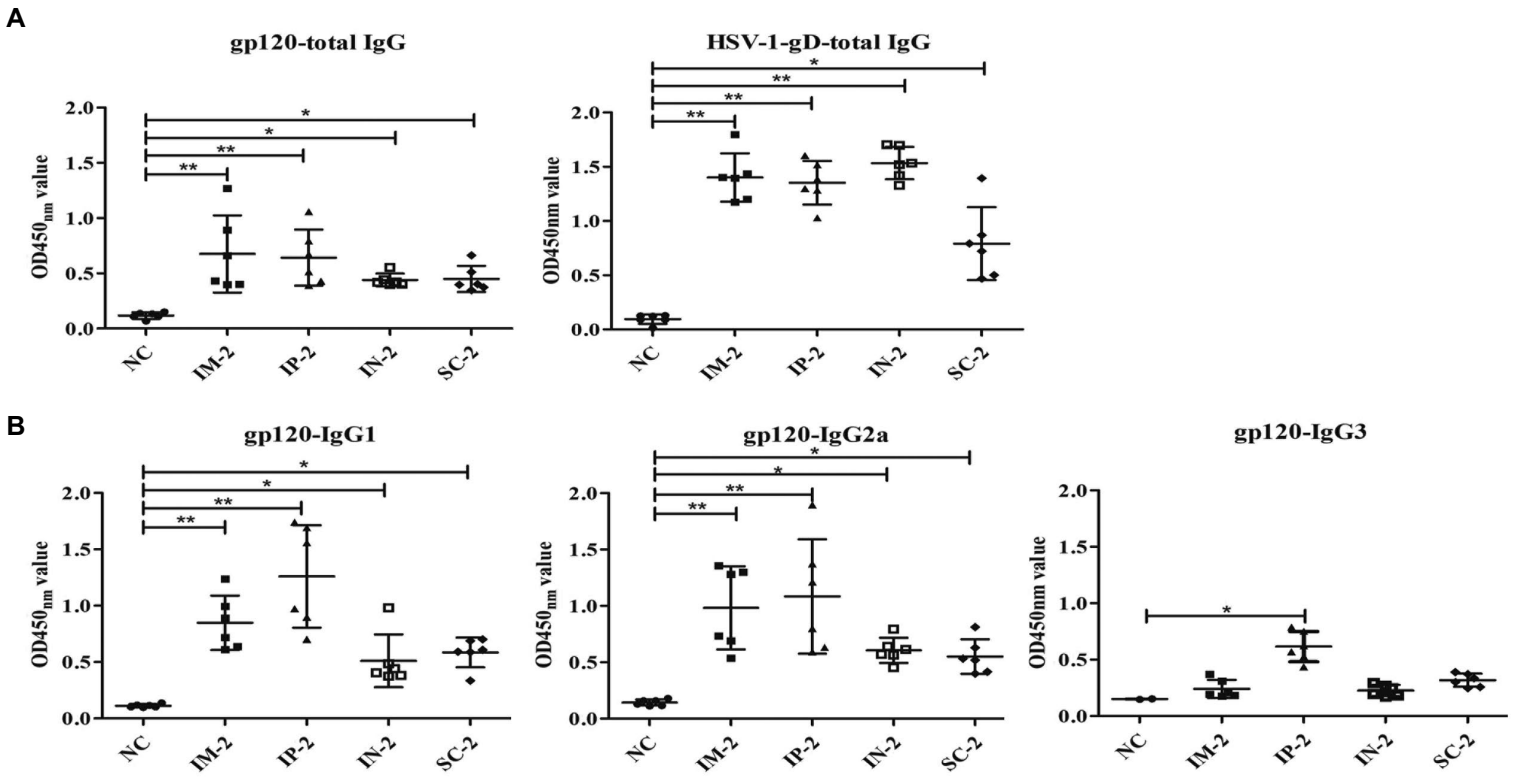
Antibody responses to different administration routes. Serum samples were collected from mice following different immunization routes 56 days after the first injection, and antigen specific humoral immune responses measured by ELISA. Pre-immune sera of each mouse were used as negative control. Serum dilution is 1:10^5^ for HSV-1 gD, and 1:10^3^ for all other antigens. **(A)** Total IgG responses specifically against gp120 and HSV-1 gD. **(B)** IgG subtype antibodies against gp120, including IgG1, IgG2a, and IgG3. Mock-infected mouse served as the negative control (NC). *p < 0.05, **p < 0.001.

To further evaluate specific serum antibody levels, ELISA was performed with gp120 and HSV-1 gD protein, respectively. As shown in Figure 4A, all four immunized groups exhibited high levels of HSV-specific and HIV-specific gp120 total IgG antibodies. In addition, serum IgG1 and IgG2a responses specific to gp120 were measured by isotype-specific ELISA (Figure 4B), the IgG1 response was strongest in the IP-2 group followed by IM-2, SC-2 and IN-2 groups, but there is no significant difference among all groups (*p* > 0.05). The IgG2a response was also strongest in the IP-2 group followed by IM-2, SC-2 and IN-2 groups. We also calculated the ratio of serum IgG1:IgG2a to assess the Th1/Th2 balance. The ratio for the IP-2 group was 1.11 and the ratios for IM-2, SC-2 and IN-2 were 0.89, 0.80 and 0.87 respectively, suggesting mixed Th1/Th2 responses, especially the IP-2 group. In addition, the IP-2 group also showed significantly higher IgG3 antibody levels (*p* < 0.05). Therefore, IP injection is the best administration route for the recombinant virus in subsequent experiments.

### T responses elicited by different antigens and vaccine regimens

**Figure 5:**
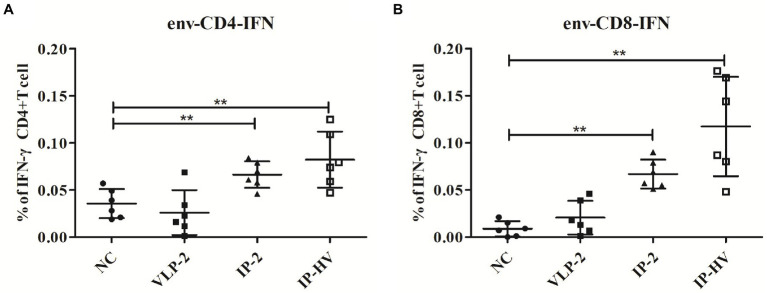
HIV-1 envelope-specific CD4 and CD8 T cell responses elicited by different immunization regimens. 56 days after the first injection, mice in different vaccination groups (VLP-2, IP-2, IP-HV and NC group) were humanly euthanized and spleens collected, T cell responses against HIV-1 envelop-specific peptides were analyzed by flow cytometry. The percentages of activated CD4^+^ **(A)** and CD8^+^ **(B)** T cells that produce IFN-γ were detected by intracellular cytokine staining. Mock-infected mouse served as the negative control (NC). *p < 0.05, **p < 0.001.

To test which antigen combination could trigger the strongest immunization responses, splenocytes from IP-2, IP-HV, VLP-2 prime-boost and control mice were tested against envelope peptide mixtures by intracellular IFN-γ cytokine staining (Figure 5). T cells from IP-HV prime-boost mice exhibited statistically significantly higher peptide-specific CD4 and CD8 T cells responses against envelope peptides (*p* < 0.05), which were better than VLP-2 and IP-2 mice. In addition, T cells from VLP-2 prime-boost mice showed the worst IFN-γ stimulating, comparable to the negative control. Interestingly, the prime-boost platform-based immunization strategy of a single antigen component (IP-2 and VLP-2) is inferior to the mixed antigen (IP-HV).

### B responses induced by different antigens and administration manners

**Figure 6:**
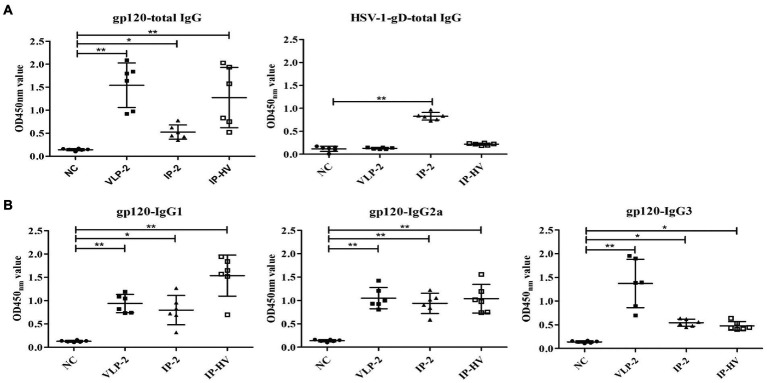
Antibody responses elicited by different immunization regimens. The serum samples were collected from mice following different immunization regimens 56 days after the first injection and antigen specific humoral immune responses measured by ELISA. Pre-immune sera of each mouse were used as negative control. Serum dilution is 1:10^5^ for HSV-1 gD, and 1:10^3^ for all other antigens. **(A)** Total IgG responses specifically against gp120 (a) and HSV-1 gD. **(B)** IgG subtype antibodies against gp120, including IgG1, IgG2a, and IgG3. Mock-infected mouse servs as the negative control (NC). *p < 0.05, **p < 0.001.

Since the IP-HV administration regimen showed superiority over other immunization groups in T cells stimulation, we next assessed the humoral immune responses. ELISA assay indicated that mice in the VLP-2 group exhibited the highest levels of HSV-specific total IgG antibodies, but mice in the IP-2 group showed the best performance when evaluating the HIV-1-gD specific total IgG antibodies (Figure 6A), and the induction of IgG, IgG2a and IgG3 were also best among these immunized groups (Figure 6B). However, except for IgG3, the antibody levels of mice in the IP-HV group were comparable to those in the VLP-2 group (*p* > 0.05), especially the IgG1 antibody. Furthermore, the ratio of serum IgG1:IgG2a from the IP-HV group (1.48) was significant lower (*p* < 0.05) than VLP-2 group (0.89), indicating that the IP-HV group mice developed a stronger TH1-type immune response, which is essential for virus clearance (Aleebrahim-Dehkordi et al., 2022).

## Discussion

The HSV-1-based vaccine is an important vector backbone for HIV-1 study (Duke et al., 2007; Nicoli et al., 2016; Polpitiya Arachchige et al., 2018). Previously we have constructed a recombinant HSV-1 virus MH1001. The virulence of MH1001 is substantially weakened by deletions of the IR sequences, resulting in inhibition of expression of specific genes, without impacting replication. Meanwhile, it is reported that the deletion of 15 kb IR of NV1020 viruses was replicated efficiently in transformed cells (Wong et al., 2001). A potentially important benefit of the MH1001 attenuation strategy is the retention of one intact 34.5 gene copy, which may broaden its efficacy and applicability in the development of vaccines (Geevarghese et al., 2010). Additionally, the safety evaluation of MH1001 did not show any signs of toxicity in mice, and all remained healthy up to the final immunization protocol (unpublished data). Therefore, recombinant virus derived from MH1001 or similar attenuated virus vector may provide lifelong latency for continuous HIV antigen delivery and immune (Marconi et al., 2009; Bloom et al., 2018). Interestingly, our work shows that HIV-1 antigen gp160 carried by the recombinant HSV-1 virus vaccine here has the characteristics of continuous and large expression, which is just located outside the cell membrane, laying the foundation for antigen presentation and vaccine function (Neukirch et al., 2020). The recombinant HSV-1 virus with HIV gp160 showed the expected immune response in mice, especially after the optimized immunization route of IP and in combination with VLPs.

In the search for an effective vaccine for HIV, many methods are being considered. The virus-derived vaccines employ a wide variety of vector systems (Egan et al., 2004; Ramsburg et al., 2004; Colby et al., 2020). Among them, the ability of herpesviruses to persist and induce durable immune responses in their infected hosts makes them an attractive viral vector (Isshiki et al., 2014); and most of vaccine evaluations are carried out non-human primates due to the limitations of HIV infected species. Primate showed promising cellular and humoral immune responses after administration of recombinant HSV-1 vectors that express key viral antigens, which were sufficient to drive viral clearance (Murphy et al., 2000). However, the evaluation of vaccine effectiveness is also conducted in murine model due to the advantages of cost-saving, operability and comparable immune response with primates (Duke et al., 2007; Kanda et al., 2016; Polpitiya Arachchige et al., 2018). Notably, heterologous DNA-VLP prime-boost enhanced immunogenicity and elicited a superior neutralizing antibody response (Ding et al., 2011; Yang et al., 2012). Moreover, vaccination-induced protection is also closely related to age, sex and genetic differences. Administration routes are one of many factors often assessed to optimize vaccine delivery (Zimmermann and Curtis, 2019). Here, mice immunized by IM, IP, IN and SC routes with recombinant viruses exhibited HIV-1 specific response, but the IP route exhibited higher cellular and humoral immune responses when compared with the other groups. Therefore, in this study the immunogenicity of recombinant viruses was evaluated in the heterologous HSV-VLP prime boost though IP administration. Specifically, the proportion of IFN-γ positive CD4^+^ and CD8^+^ T cells averaged 0.11% following HSV-VLP prime-boost i.p. immunization (IP-HV), while the average proportion of VLP-2 mice was 0.02% and much lower than those expressed by HSV-1 vectors (IP-HV and IP-2), as expected (Parker et al., 2007). The humoral immune response detected in our studies mainly involved IgG1 and IgG2a subclasses induced by HSV-1. Thus, the HSV-1 vector appears to elicit a mixed Th1/Th2 response, and VLP boosting promotes Th1. The lower IgG3 and neutralizing antibody responses for HSV-1 viruses could be due to the virus-based vectors and the interference of anti-vector antibodies (Cooney et al., 1991; Kostense et al., 2004).

Many vector systems are being developed for use in vaccine design (Bloom et al., 2018; Excler and Kim, 2019), but prior immunity to each vector may potentially interfere with initial vaccine responses and/or booster doses required to maintain host immunity. The pre-existing immunity of HSV vectors has been investigated in mice, with conflicting results. It has been reported that HSV recombinants elicit antigen-specific immune response despite pre-existing immunity against viral antigens in the host (Pushko et al., 1997; Kuklin et al., 1998; Brockman and Knipe, 2002). However, studies in mice show anti-HSV-1 antibodies significant declined humoral and cell-associated immune responses after vaccination of manually modified HSV vaccine when compared with HSV-1 immunized naïve mice (Lauterbach et al., 2005). Mice receiving second immunization with HSV-1 viruses were affected by the prior HSV-1 immunity, resulting in attenuated cellular response when compared to the single injection group. These results are consistent with previous studies (Desrosiers, 2004). Interestingly, prime-boost strategy with heterotypic antigen (IP-HIV) greatly improved the immune response surpassing the single use of recombinant virus vector or protein in this study. The combination of heterologous immunogens shows superiority in all detection indexes of immune response including CD4^+^ and CD8^+^ T-cells and IgG antibodies.

Collectively, recombinant HSV-1 viruses can induce specific immune responses against HIV-1 in immunized animals, and IP vaccination demonstrates preferential immune induction. Additionally, the combined immunization of the virus and VLP outperforms the humoral and cellular immune response raised against a single immune virus or VLP in the prime-boost platform. Of note, the mixed immunization mode of different immunogens offset the immunosuppressive effect caused by single antigen immunization. Thus, combinatory DNA-protein vaccination regimens are a promising alternative for HIV prevention and should be considered for all vaccine candidates.

## Conclusion

HSV-1-based virus vector is widely used in the vaccine development carrying HIV genes that provide immune protection to immunized individuals. Here, expression of the target gene gp160 of HIV-1 in the HSV-1 system effectively elicited a comprehensive immune response. It is worth noting that prime-boost with two different immunogens (virus and protein) was much better than each one individually, especially in stimulation of T cell immune response.

## Data availability statement

The original contributions presented in the study are included in the article/supplementary material, further inquiries can be directed to the corresponding author.

## Ethics statement

The animal study was reviewed and approved by the Committee on Ethical Use of Animals of Xinjiang University.

## Author contributions

ZM and PZ conceptualized and designed the experiments. BZ and HM performed the experiments. BZ, HM, JG, and ZM analyzed the data. BZ was responsible for writing-original draft preparation. ZM and PZ were responsible for writing-reviewing and editing. All authors contributed to the article and approved the submitted version.

## Funding

This work was supported by grants from the Xinjiang Uygur autonomous region High-tech Research and Development Project (2010016) to ZM and the Youth Fund of Natural Science Foundation of Xinjiang Uygur Autonomous Region (No. 2022D01C697) awarded to BZ.

## Conflict of interest

The authors declare that the research was conducted in the absence of any commercial or financial relationships that could be construed as a potential conflict of interest.

## Publisher’s note

All claims expressed in this article are solely those of the authors and do not necessarily represent those of their affiliated organizations, or those of the publisher, the editors and the reviewers. Any product that may be evaluated in this article, or claim that may be made by its manufacturer, is not guaranteed or endorsed by the publisher.

## Footnotes

^1^ https://www.who.int/home

## References

[ref1] Abendroth A. Morrow G. Cunningham A. L. Slobedman B. (2001). Varicella-zoster virus infection of human dendritic cells and transmission to T cells: implications for virus dissemination in the host. J. Virol. 75, 6183–6192. doi: 10.1128/jvi.75.13.6183-6192.2001, PMID: 11390620 PMC114334

[ref2] Aleebrahim-Dehkordi E. Molavi B. Mokhtari M. Deravi N. Fathi M. Fazel T.. (2022). T helper type (Th1/Th2) responses to SARS-CoV-2 and influenza A (H1N1) virus: from cytokines produced to immune responses. Transpl. Immunol. 70, 101495–101504. doi: 10.1016/j.trim.2021.101495, PMID: 34774738 PMC8579696

[ref3] Altfeld M. Gale M. Jr. (2015). Innate immunity against HIV-1 infection. Nat. Immunol. 16, 554–562. doi: 10.1038/ni.3157 25988887

[ref4] Ayele G. Tessema B. Amsalu A. Ferede G. Yismaw G. (2018). Prevalence and associated factors of treatment failure among HIV/AIDS patients on HAART attending University of Gondar Referral Hospital Northwest Ethiopia. BMC Immunol. 19:37. doi: 10.1186/s12865-018-0278-4, PMID: 30558580 PMC6296084

[ref5] Barre-Sinoussi F. Ross A. L. Delfraissy J. F. (2013). Past, present and future: 30 years of HIV research. Nat. Rev. Microbiol. 11, 877–883. doi: 10.1038/nrmicro3132, PMID: 24162027

[ref6] Bloom D. C. Tran R. K. Feller J. Voellmy R. (2018). Immunization by replication-competent controlled herpesvirus vectors. J. Virol. 92:e00616-18. doi: 10.1128/JVI.00616-18, PMID: 29899091 PMC6069180

[ref7] Brehm M. Samaniego L. A. Bonneau R. H. DeLuca N. A. Tevethia S. S. (1999). Immunogenicity of herpes simplex virus type 1 mutants containing deletions in one or more α-genes: ICP4, ICP27, ICP22, and ICP0. Virology 256, 258–269. doi: 10.1006/viro.1999.9653, PMID: 10191191

[ref8] Brockman M. A. Knipe D. M. (2002). Herpes simplex virus vectors elicit durable immune responses in the presence of preexisting host immunity. J. Virol. 76, 3678–3687. doi: 10.1128/jvi.76.8.3678-3687.2002, PMID: 11907207 PMC136066

[ref9] Cafaro A. Tripiciano A. Sgadari C. Bellino S. Picconi O. Longo O.. (2015). Development of a novel AIDS vaccine: the HIV-1 transactivator of transcription protein vaccine. Expert. Opin. Biol. Ther. 15, 13–29. doi: 10.1517/14712598.2015.1021328, PMID: 26096836

[ref10] Calarota S. A. Weiner D. B. (2003). Present status of human HIV vaccine development. AIDS 17, S73–S84. doi: 10.1097/00002030-200317004-00009 15080183

[ref11] Colby D. J. Sarnecki M. Barouch D. H. Tipsuk S. Stieh D. J. Kroon E.. (2020). Safety and immunogenicity of Ad26 and MVA vaccines in acutely treated HIV and effect on viral rebound after antiretroviral therapy interruption. Nat. Med. 26, 498–501. doi: 10.1038/s41591-020-0774-y, PMID: 32235883

[ref12] Cooney E. L. Collier A. C. Greenberg P. D. Coombs R. W. Zarling J. Arditti D. E.. (1991). Safety of and immunological response to a recombinant vaccinia virus vaccine expressing HIV envelope glycoprotein. Lancet 337, 567–572. doi: 10.1016/0140-6736(91)91636-9, PMID: 1671940

[ref13] Desrosiers R. C. (1999). Strategies used by human immunodeficiency virus that allow persistent viral replication. Nat. Med. 5, 723–725. doi: 10.1038/10439 10395309

[ref14] Desrosiers R. C. (2004). Prospects for an AIDS vaccine. Nat. Med. 10, 221–223. doi: 10.1038/nm0304-221 14991035

[ref15] Ding H. Tsai C. Gutiérrez R. A. Zhou F. Buchy P. Deubel V.. (2011). Superior neutralizing antibody response and protection in mice vaccinated with heterologous DNA prime and virus like particle boost against HPAI H5N1 virus. PLoS One 6:e16563. doi: 10.1371/journal.pone.0016563, PMID: 21305045 PMC3030595

[ref16] Duke C. M. Maguire C. A. Keefer M. C. Federoff H. J. Bowers W. J. Dewhurst S. (2007). HSV-1 amplicon vectors elicit polyfunctional T cell responses to HIV-1 Env, and strongly boost responses to an adenovirus prime. Vaccine 25, 7410–7421. doi: 10.1016/j.vaccine.2007.08.015, PMID: 17868958 PMC2092414

[ref17] Egan M. A. Chong S. Y. Rose N. F. Megathi S. Lopez K. J. Schadeck E. B.. (2004). Immunogenicity of attenuated vesicular stomatitis virus vectors expressing HIV type 1 Env and SIV gag proteins: comparison of intranasal and intramuscular vaccination routes. AIDS Res. Hum. Retrovir. 20, 989–1004. doi: 10.1089/aid.2004.20.989, PMID: 15585086

[ref18] Emini E. A. Koff W. C. (2004). Developing an AIDS vaccine: need, uncertainty, hope. Science. 304, 1913–1914. doi: 10.1126/science.1100368, PMID: 15218131

[ref19] Excler J. L. Kim J. H. (2019). Novel prime-boost vaccine strategies against HIV-1. Expert Rev. Vaccines 18, 765–779. doi: 10.1080/14760584.2019.1640117, PMID: 31271322

[ref20] Geevarghese S. K. Geller D. A. de Haan H. A. Hörer M. Knoll A. E. Mescheder A.. (2010). Phase I/II study of oncolytic herpes simplex virus NV1020 in patients with extensively pretreated refractory colorectal cancer metastatic to the liver. Hum. Gene Ther. 21, 1119–1128. doi: 10.1089/hum.2010.020, PMID: 20486770 PMC3733135

[ref21] Graziani G. M. Angel J. B. (2016). HIV-1 immunogen: an overview of almost 30 years of clinical testing of a candidate therapeutic vaccine. Expert. Opin. Biol. Ther. 16, 953–966. doi: 10.1080/14712598.2016.1193594, PMID: 27266543

[ref22] Haglund K. Leiner I. Kerksiek K. Buonocore L. Pamer E. Rose J. K. (2002). Robust recall and long-term memory T-cell responses induced by prime-boost regimens with heterologous live viral vectors expressing human immunodeficiency virus type 1 Gag and Env proteins. J. Virol. 76, 7506–7517. doi: 10.1128/jvi.76.15.7506-7517.2002, PMID: 12097563 PMC136360

[ref23] Hocknell P. K. Wiley R. D. Wang X. Evans T. G. Bowers W. J. Hanke T.. (2002). Expression of human immunodeficiency virus type 1 gp120 from herpes simplex virus type 1-derived amplicons results in potent, specific, and durable cellular and humoral immune responses. J. Virol. 76, 5565–5580. doi: 10.1128/jvi.76.11.5565-5580.2002, PMID: 11991985 PMC137011

[ref24] Hollister K. Chen Y. Wang S. Wu H. Mondal A. Clegg N.. (2014). The role of follicular helper T cells and the germinal center in HIV-1 gp120 DNA prime and gp120 protein boost vaccination. Hum. Vaccin. Immunother. 10, 1985–1992. doi: 10.4161/hv.28659, PMID: 25424808 PMC4186047

[ref25] Horsburgh B. C. Hubinette M. M. Tufaro F. (1999). Genetic manipulation of herpes simplex virus using bacterial artificial chromosomes. Methods Enzymol. 306, 337–352. doi: 10.1016/s0076-6879(99)06022-x, PMID: 10432464

[ref26] Isshiki M. Zhang X. Sato H. Ohashi T. Inoue M. Shida H. (2014). Effects of different promoters on the virulence and immunogenicity of a HIV-1 Env-expressing recombinant vaccinia vaccine. Vaccine 32, 839–845. doi: 10.1016/j.vaccine.2013.12.022, PMID: 24370703

[ref27] James C. Harfouche M. Welton N. J. Turner K. M. Abu-Raddad L. J. Gottlieb S. L.. (2020). Herpes simplex virus: global infection prevalence and incidence estimates, 2016. Bull. World Health Organ. 98, 315–329. doi: 10.2471/BLT.19.237149, PMID: 32514197 PMC7265941

[ref28] Kanda H. Kanao M. Liu S. Yi H. Iida T. Levitt R. C.. (2016). HSV vector-mediated GAD67 suppresses neuropathic pain induced by perineural HIV gp120 in rats through inhibition of ROS and Wnt5a. Gene Ther. 23, 340–348. doi: 10.1038/gt.2016.3, PMID: 26752351 PMC4824655

[ref29] Kaur A. Sanford H. B. Garry D. Lang S. Klumpp S. A. Watanabe D.. (2007). Ability of herpes simplex virus vectors to boost immune responses to dna vectors and to protect against challenge by simian immunodeficiency virus. Virology 357, 199–214. doi: 10.1016/j.virol.2006.08.007, PMID: 16962628 PMC1819472

[ref30] Kostense S. Koudstaal W. Sprangers M. Weverling G. J. Penders G. Helmus N.. (2004). Adenovirus types 5 and 35 seroprevalence in AIDS risk groups supports type 35 as a vaccine vector. AIDS 18, 1213–1216. doi: 10.1097/00002030-200405210-00019, PMID: 15166541

[ref31] Kruse M. Rosorius O. Kratzer F. Stelz G. Kuhnt C. Schuler G.. (2000). Mature dendritic cells infected with herpes simplex virus type 1 exhibit inhibited T-cell stimulatory capacity. J. Virol. 74, 7127–7136. doi: 10.1128/jvi.74.15.7127-7136.2000, PMID: 10888653 PMC112231

[ref32] Kuklin N. A. Daheshia M. Marconi P. C. Krisky D. M. Rouse R. J. D. Glorioso J. C.. (1998). Modulation of mucosal and systemic immunity by enteric administration of nonreplicating herpes simplex virus expressing cytokines. Virology 240, 245–253. doi: 10.1006/viro.1997.8926, PMID: 9454698

[ref33] Kuroda T. Martuza R. L. Todo T. Rabkin S. D. (2006). Flip-Flop HSV-BAC: bacterial artificial chromosome based system for rapid generation of recombinant herpes simplex virus vectors using two independent site-specific recombinases. BMC Biotechnol. 6, 40–58. doi: 10.1186/1472-6750-6-40, PMID: 16995942 PMC1609115

[ref34] Larijani M. S. Ramezani A. Sadat S. M. (2019). Updated studies on the development of HIV therapeutic vaccine. Curr. HIV Res. 17, 75–84. doi: 10.2174/1570162X17666190618160608, PMID: 31210114

[ref35] Lauterbach H. Ried C. Epstein A. L. Marconi P. Brocker T. (2005). Reduced immune responses after vaccination with a recombinant herpes simplex virus type 1 vector in the presence of antiviral immunity. J. Gen. Virol. 86, 2401–2410. doi: 10.1099/vir.0.81104-0, PMID: 16099897

[ref36] Li J. Li J. Aipire A. Luo J. Yuan P. Zhang F. (2016). The combination of *Pleurotus ferulae* water extract and CpG-ODN enhances the immune responses and antitumor efficacy of HPV peptides pulsed dendritic cell-based vaccine. Vaccine 34, 3568–3575. doi: 10.1016/j.vaccine.2016.05.022, PMID: 27211038

[ref37] Liu X. Broberg E. Watanabe D. Dudek T. Deluca N. Knipe D. M. (2009). Genetic engineering of a modified herpes simplex virus 1 vaccine vector. Vaccine 27, 2760–2767. doi: 10.1016/j.vaccine.2009.03.003, PMID: 19428888 PMC2680798

[ref38] Mao H. Zhao X. Zhu H. Guo J. Ma Z. (2016). Expression and immunogenicity of recombinant glycoprotein D of herpes simplex virus 1 in drosophila S2 cells. Prep. Biochem. Biotechnol. 46, 384–391. doi: 10.1080/10826068.2015.1045610, PMID: 26835587

[ref39] Marconi P. Argnani R. Epstein A. L. Manservigi R. (2009). HSV as a vector in vaccine development and gene therapy. Adv. Exp. Med. Biol. 655, 118–144. doi: 10.1007/978-1-4419-1132-2_10, PMID: 20047039

[ref40] Miall A. McLellan R. Dong K. Ndung'u T. Saberi P. Sauceda J. A.. (2022). Bringing social context into global biomedical HIV cure-related research: an urgent call to action. J. Virus Erad. 8, 100062–100064. doi: 10.1016/j.jve.2021.100062, PMID: 35169489 PMC8829132

[ref41] Mikloska Z. Bosnjak L. Cunningham A. L. (2001). Immature monocyte-derived dendritic cells are productively infected with herpes simplex virus type 1. J. Virol. 75, 5958–5964. doi: 10.1128/jvi.75.13.5958-5964.2001, PMID: 11390597 PMC114311

[ref42] Murphy C. G. Lucas W. T. Means R. E. Czajak S. Hale C. L. Lifson J. D.. (2000). Vaccine protection against simian immunodeficiency virus by recombinant strains of herpes simplex virus. J. Virol. 74, 7745–7754. doi: 10.1128/jvi.74.17.7745-7754.2000, PMID: 10933680 PMC112303

[ref43] Neukirch L. Fougeroux C. Andersson A. C. Holst P. J. (2020). The potential of adenoviral vaccine vectors with altered antigen presentation capabilities. Expert Rev. Vaccines 19, 25–41. doi: 10.1080/14760584.2020.1711054, PMID: 31889453

[ref44] Nicoli F. Gallerani E. Skarlis C. Sicurella M. Cafaro A. Ensoli B.. (2016). Systemic immunodominant CD8 responses with an effector-like phenotype are induced by intravaginal immunization with attenuated HSV vectors expressing HIV Tat and mediate protection against HSV infection. Vaccine 34, 2216–2224. doi: 10.1016/j.vaccine.2016.03.022, PMID: 27002499

[ref45] Parker S. D. Rottinghaus S. T. Zajac A. J. Yue L. Hunter E. Whitley R. J.. (2007). HIV-1(89.6) gag expressed from a replication competent HSV-1 vector elicits persistent cellular immune responses in mice. Vaccine 25, 6764–6773. doi: 10.1016/j.vaccine.2007.06.064, PMID: 17706843 PMC2084203

[ref46] Polpitiya Arachchige S. Henke W. Pramanik A. Kalamvoki M. Stephens E. B. (2018). Analysis of select herpes simplex virus 1 (HSV-1) proteins for restriction of human immunodeficiency virus type 1 (HIV-1): HSV-1 gM protein potently restricts HIV-1 by preventing intracellular transport and processing of Env gp160. J. Virol. 92:e01476-17. doi: 10.1128/JVI.01476-17, PMID: 29093081 PMC5752927

[ref47] Pushko P. Parker M. Ludwig G. V. Davis N. L. Johnston R. E. Smith J. F. (1997). Replicon-helper systems from attenuated *Venezuelan equine* encephalitis virus: expression of heterologous genes in vitro and immunization against heterologous pathogens in vivo. Virology 239, 389–401. doi: 10.1006/viro.1997.8878, PMID: 9434729

[ref48] Ramsburg E. Rose N. F. Marx P. A. Mefford M. Nixon D. F. Moretto W. J.. (2004). Highly effective control of an AIDS virus challenge in macaques by using vesicular stomatitis virus and modified vaccinia virus Ankara vaccine vectors in a single-boost protocol. J. Virol. 78, 3930–3940. doi: 10.1128/jvi.78.8.3930-3940.2004, PMID: 15047809 PMC374300

[ref49] Rerks-Ngarm S. Pitisuttithum P. Nitayaphan S. Kaewkungwal J. Chiu J. Paris R.. (2009). Vaccination with ALVAC and AIDSVAX to prevent HIV-1 infection in Thailand. N. Engl. J. Med. 361, 2209–2220. doi: 10.1056/NEJMoa0908492, PMID: 19843557

[ref50] Rose N. F. Marx P. A. Luckay A. Nixon D. F. Moretto W. J. Donahoe S. M.. (2001). An effective AIDS vaccine based on live attenuated vesicular stomatitis virus recombinants. Cells 106, 539–549. doi: 10.1016/S0092-8674(01)00482-2, PMID: 11551502

[ref51] Santos K. Duke C. M. Rodriguez-Colon S. M. Dakwar A. Fan S. Keefer M. C.. (2007). Effect of promoter strength on protein expression and immunogenicity of an HSV-1 amplicon vector encoding HIV-1 Gag. Vaccine 25, 1634–1646. doi: 10.1016/j.vaccine.2006.11.004, PMID: 17145123 PMC1851942

[ref52] Spearman P. (2003). HIV vaccine development: lessons from the past and promise for the future. Curr. HIV Res. 1, 101–120. doi: 10.2174/1570162033352093, PMID: 15043215

[ref53] Spivack J. G. Fareed M. U. Valyi-Nagy T. Nash T. C. O'Keefe J. S. Gesser R. M.. (1995). Replication, establishment of latent infection, expression of the latency-associated transcripts and explant reactivation of herpes simplex virus type 1 γ34.5 mutants in a mouse eye model. J. Gen. Virol. 76, 321–332. doi: 10.1099/0022-1317-76-2-321 7844554

[ref54] Uhl E. W. Heaton-Jones T. G. Pu R. Yamamoto J. K. (2002). FIV vaccine development and its importance to veterinary and human medicine: a review: FIV vaccine 2002 update and review. Vet. Immunol. Immunopathol. 90, 113–132. doi: 10.1016/S0165-2427(02)00227-1, PMID: 12459160 PMC7119750

[ref55] Verrier B. Le Grand R. Ataman-Önal Y. Terrat C. Guillon C. Durand P.-Y.. (2002). Evaluation in rhesus macaques of Tat and rev-targeted immunization as a preventive vaccine against mucosal challenge with SHIV-BX08. DNA Cell Biol. 21, 653–658. doi: 10.1089/104454902760330183, PMID: 12396607

[ref56] Wang X. Zhang G. R. Sun M. Geller A. I. (2001). General strategy for constructing large HSV-1 plasmid vectors that co-express multiple genes. BioTechniques 31, 204–212. doi: 10.2144/01311dd05, PMID: 11464513 PMC2581878

[ref57] Watanabe D. Brockman M. A. Ndung'u T. Mathews L. Lucas W. T. Murphy C. G.. (2007). Properties of a herpes simplex virus multiple immediate-early gene-deleted recombinant as a vaccine vector. Virology 357, 186–198. doi: 10.1016/j.virol.2006.08.015, PMID: 16996101

[ref58] Whitley R. J. Kern E. R. Chatterjee S. Chou J. Roizman B. (1993). Replication, establishment of latency, and induced reactivation of herpes simplex virus gamma 1 34.5 deletion mutants in rodent models. J. Clin. Invest. 91, 2837–2843. doi: 10.1172/JCI116527, PMID: 8390490 PMC443352

[ref59] Wong R. J. Kim S.-H. Joe J. K. Shah J. P. Johnson P. A. Fong Y. (2001). Effective treatment of head and neck squamous cell carcinoma by an oncolytic herpes simplex virus1. J. Am. Coll. Surg. 193, 12–21. doi: 10.1016/S1072-7515(01)00866-3, PMID: 11442249

[ref60] Woodman Z. Williamson C. (2009). HIV molecular epidemiology: transmission and adaptation to human populations. Curr. Opin. HIV AIDS 4, 247–252. doi: 10.1097/COH.0b013e32832c0672, PMID: 19532060 PMC3351078

[ref61] Xiao P. Dienger-Stambaugh K. Chen X. Wei H. Phan S. Beavis A. C.. (2021). Parainfluenza virus 5 priming followed by SIV/HIV virus-like-particle boosting induces potent and durable immune responses in nonhuman primates. Front. Immunol. 12, 623996–6234010. doi: 10.3389/fimmu.2021.623996, PMID: 33717130 PMC7946978

[ref62] Yang L. Song Y. Li X. Huang X. Liu J. Ding H.. (2012). HIV-1 virus-like particles produced by stably transfected *Drosophila* S2 cells: a desirable vaccine component. J. Virol. 86, 7662–7676. doi: 10.1128/JVI.07164-11, PMID: 22553333 PMC3416305

[ref63] Zimmermann P. Curtis N. (2019). Factors that influence the immune response to vaccination. Clin. Microbiol. Rev. 32:e00084-18. doi: 10.1128/CMR.00084-18, PMID: 30867162 PMC6431125

